# Advances in the Dereplication of Aroma Precursors from Grape Juice by Pretreatment with Lead Acetate and Combined HILIC- and RP-HPLC Methods

**DOI:** 10.3390/foods8010028

**Published:** 2019-01-15

**Authors:** Michele D’Ambrosio

**Affiliations:** Laboratory of Bio-Organic Chemistry, Department of Physics, University of Trento, via Sommarive 14, 38123 Trento, Italy; michele.dambrosio@unitn.it; Tel.: +39-461-281-509

**Keywords:** aroma precursors, monoterpene glycoside, grape, wine, HILIC, NMR, lead acetate

## Abstract

Glycosidic aroma precursors (GAPs) contribute to the varietal flavor of wine. Researchers have applied various sample preparation and analytical methods in attempts to achieve their separation and identification. However, mass spectrometric methods still fail to unequivocally define their structures. We have previously reported the separation of GAPs in their natural form and elucidated their structures by nuclear magnetic resonance (NMR) spectroscopy. In this study, we confirm the effectiveness of our established procedure and present methodological improvements. Grape juice was treated with lead (II) acetate and repeatedly chromatographed to give seven pure GAPs. Their chemical structures were characterized by MS^n^ fragmentations and 1D- and 2D-NMR spectra. Ten GAPs were analyzed by both hydrophilic interaction liquid chromatography (HILIC) and reversed phase high performance liquid chromatography (RP-HPLC) to compare the two chromatograms. A selection of known phenols was treated with lead (II) acetate in order to check its binding properties.

## 1. Introduction

The flavor precursors present in grapes and wine comprise a heterogeneous blend of mono- and disaccharide glycosides of volatile aglycones, which include monoterpenes, C_13_-norisoprenoids, benzene derivatives, and long-chain aliphatic alcohols [1]. The compositional analysis of glycoconjugates has been undertaken by several procedures [2].

At the analytical level, a method using Fourier-transform infrared (FTIR) spectrometry and chemometric techniques allowed the rapid determination of C_13_-norisoprenoidic and monoterpene glycoconjugates, with predictive errors of 14% and 15%, respectively [3]. No improvement seems to have been published to date. Early experiments carried out the identification of glycoconjugates by exhaustive trifluoroacetylation of sugar hydroxyls followed by GC-MS analysis and subsequent comparisons of retention times (*t_R_*) and mass spectra with synthetic standards [4]. Recently, the high sensitivity and selectivity of the high-performance liquid chromatography (HPLC) system interfaced to the high-resolution mass spectrometry (HRMS) unit was exploited to characterize the GAPs in grapes [5]. The experiments disclosed 20 monoterpene-diglycoside derivatives but could not confirm the structure of isobaric aglycones and their sugar residues.

At the preparative level, liquid chromatography is the recommended analytical technique. Countercurrent chromatography has been widely used and its applications reviewed [6]. However, even in this case, purification still requires peracetylation of the glycosidic compounds. HPLC purification using reverse phase (RP) columns offers an effective alternative and this approach allowed isolation of 12 GAPs from “Moscato Giallo” grapes [7]. Hydrophilic interaction liquid chromatography (HILIC) is a technique that uses a hydrophilic stationary phase and an aqueous–organic solvent as the mobile phase. HILIC provides a particularly powerful method for the separation of polar compounds and it has been successfully applied to the isolation of aroma precursors [8]. 

The treatment of grape juice with lead (II) acetate precipitates phenol compounds such as cinnamic acid derivatives, flavonoids, and anthocyanins, without modifying the chemical structure of flavor glycoconjugates. This isolation protocol could be optimized by combining it with the HILIC method in sequence with reversed phase (RP) elution. This study therefore aimed to: (1) isolate and elucidate GAPs from “Moscato Rosa” grapes; (2) investigate the differences and synergies between HILIC and RP-HPLC stationary phases; and (3) confirm the effectiveness of lead acetate in precipitating phenols.

## 2. Materials and Methods

### 2.1. Chemicals

Analytical grade solvents were used for extraction and flash chromatography (FC); ethanol was HPLC-grade (VWR International, Fontenay-sous-Bois, France). Amberlite^®^ XAD-2 resin was purchased from Fluka Chemie (Büchs, Switzerland) and lead acetate Pb(OAc)_2_ from Merck (Steinheim, Germany). Two stationary phases, cyanopropyl silica (CN, LiChroprep^®^ CN 40–63 µm, Merck, Darmstadt, Germany) and octadecyl silica (ODS, LiChroprep^®^ RP-18 40–63 µm, Merck, Darmstadt, Germany), were employed for FC.

### 2.2. Apparatus and Method

The preparative HPLC system consisted of a Merck Hitachi model L-7100 pump, an L-7400 UV detector, a D-7500 integrator, and a Rheodyne manual injector equipped with a 200 µL loop. Analytical separations were carried out using an Agilent 1100 series LC system consisting of a binary pump, a vacuum degasser, an autosampler with standard analytical head (100 µL), a column thermostat, and a 1200 series diode array detector (DAD) and evaporative light scattering detector (ELSD). The column oven temperature was fixed at 25 °C and the UV or DAD detectors were set at λ = 210 nm. The ELSD was connected in series with the DAD; the drift tube temperature was set at 50 °C and the pressure of nitrogen gas at 3.5 bar.

The analyses of components in RP mode were performed on a Synergi Hydro column (150 × 4.6 mm or 150 × 10.0 mm, 4 µm, 80 Å; Phenomenex, Torrance, CA, USA). The HILIC column was a ZIC SeQuant (150 × 10 mm, 5 µm, 200 Å; Merck). The injection volume was 10 µL. 

Nuclear magnetic resonance (NMR) spectra were acquired using a Bruker Avance 400 spectrometer (^1^H at 400 MHz) and a 5 mm BBI probe. Chemical shifts were reported in ppm on the δ scale with the residual solvent signal used as an internal reference (CD_3_OD = 49.0, CD_2_HOD = 3.31, DHO = 4.72), proton coupling constants (J values in Hz) and multiplicities from 1D spectra, peak assignments from ^1^H,^1^H-COSY, and ^1^J_CH_ (edited HSQC) and ^n^J_CH_ (HMBC) heterocorrelation experiments. NOESY data are reported as correlation map(s) between protons ^1^H ↔ ^1^H; HMBC data are reported as ^13^C→correlated to ^1^H. 

LC-MS analyses were performed using an Agilent 1100 series LC system interfaced to a Bruker model Esquire_LC multiple ion trap mass spectrometer equipped with an atmospheric pressure interface electrospray (API-ES) chamber. Conditions for electrospray ionization mass spectrometry (ESI-MS) analysis of HPLC peaks in both positive and negative ion mode included a capillary voltage of 4000 V, a nebulizing pressure of 30.0 psi, a drying gas flow of 7 mL/min, and a temperature of 300 °C. 

### 2.3. Plant Material

*Vitis vinifera* L. cultivar “Moscato Rosa” grapes (5.8 kg) were harvested in 2012 at the vineyard of Azienda Agricola ZENI, Sorni di Lavis, Trentino. After harvesting, the grapes (~35.0 °Brix) were crushed and sodium metabisulfite Na_2_S_2_O_5_ (300 mg/L) was added to the juice, which was promptly extracted.

### 2.4. Sample Preparation

Fresh grape juice (2 L) was centrifuged at 3000 rpm for 15 min at room temperature (RT). The supernatant (in batches of ~1 L) was passed three times through a column (25 cm × 4 cm i.d.) packed with Amberlite^®^ XAD-2. The column was washed with water (2 L) and eluted with ethanol (2 L). The alcoholic eluate was desiccated in vacuo to give ~1.4 g of adsorbed organic material.

The dry extract was subjected to flash chromatography (8 cm × 4 cm i.d.) on CN with stepwise elution of 50% ethyl acetate in hexane (100 mL, fr. 1, 88 mg), 100% ethyl acetate (300 mL, fr. 2, 410 mg), and 100% ethanol (200 mL, fr. 3, 210 mg). 

Aliquots of the pooled FC fractions 2 and 3 (190 mg) were dissolved in 6 mL of methanol and lead (II) acetate (0.8 g) was added. The mixture was stirred for two hours at RT then centrifuged at 3000 rpm for 15 min. The precipitate was washed with methanol and discarded. The ODS stationary phase (1 spoon) was added to the clear solution and the solvent was evaporated. The desiccated slurry was applied to an ODS column (8 cm × 4 cm i.d.) and chromatographed with a gradient of methanol in water (15:85, 30:70, 45:55, 60:40, 100:0). 

### 2.5. Isolation of the Glycosidic Fractions

Central fractions (40 mg), eluted with methanol ranging from 30% to 45%, were dissolved in ethanol (1 mL) and purified by RP-HPLC, with the detection wavelength set at λ = 210 nm and the flux at 5 mL/min. The mobile phase consisted of ethanol/water 1:1 (A) and water (B) applied in the following stepped linear gradient: A = 40% at *t* = 0, A = 70% at *t* = 10, A = 70% at *t* = 30, A = 100% at *t* = 40, A = 40% at *t* = 42 min, and 13 min equilibration time.

The peaks of compounds **4**, **5**, **6**, **7**, **8**, **9**, and **10** were eluted at *t_R_* = 6.0 (2.8 mg), 10.4 (1.1 mg), 18.0 (1.6 mg), 19.2 (2.9 mg), 21.1 (4.9 mg), 23.7 (1.2 mg), and 24.8 (1.5 mg) min, respectively. 

### 2.6. Comparative Chromatography of the Aroma Precursors

The analyses of components in RP mode were performed at a flux of 0.8 mL/min. The mobile phase consisted of ethanol/water 10:90 (A) and ethanol/water 50:50 (B) applied in the following stepped linear gradient: A = 90% at *t* = 0, A = 40% at *t* = 18, A = 40% at *t* = 40, A = 90% at *t* = 41 min, and 9 min as equilibration time. The analyses of components in normal phase (HILIC) mode were performed at a flux of 0.2 mL/min. The mobile phase consisted of acetonitrile (A) and water (1% *v*/*v* HCOOH) (B) applied in the following stepped linear gradient: A = 98% at *t* = 0, A = 94% at *t* = 40, A = 50% at *t* = 48, A = 50% at *t* = 55, A = 98% at *t* = 56 min, and 9 min equilibration time.

### 2.7. Precipitation of Model Phenols by Lead (II) Acetate

Three classes of compounds were selected for treatment with lead acetate: (A) simple phenols; (B) flavonoids; and (C) anthocyanins. Group A included resveratrol (0.1 mmol), cinnamic acid (0.1 mmol), coumaric acid (0.1 mmol), caffeic acid (0.1 mmol), and ferulic acid (0.1 mmol). Group B included naringin (0.025 mmol), diosmin (0.025 mmol), kaempferol (0.025 mmol), luteolin 7-*O*-glucoside (0.01 mmol), and quercetin (0.015 mmol). Group C included kaempferol 3-*O*-glucoside (0.005 mmol), cyanidin (0.01 mmol), myrtillin (0.005 mmol), and malvin (0.005 mmol). 

For each group, a stock solution (10 mL) was divided into 10 identical aliquots (1.0 mL), one of which was used as a reference. Six aliquots were then treated with lead acetate at three different concentrations (0.5, 1.0, and 2.0 molar equivalents) and two pH levels. The acidic level was represented by the initial pH value of each solution, while the basic level was obtained by adjusting three aliquots (for each group) to a pH of ~8.0 with 0.1 M aqueous NaOH.

The appropriate amount of lead acetate was dissolved in methanol (0.5 mL), then mixed with an aliquot, stirred for 15 min at RT, and finally centrifuged at 2500 rpm for 15 min. The supernatant was passed through a 0.45 mm filter, evaporated to dryness, dissolved in methanol (or methanol/water 1:1) to a volume of 1.0 mL, and chromatographed by RP-HPLC. The detection wavelength was set at λ = 280 nm. For Group A, elution started at 0.25% *v*/*v* acetonitrile in water (1% *v*/*v* FA), then acetonitrile was increased to 50% over 30 min and maintained at 50% for 10 min with the flux at 1 mL/min. For Group B, elution started at 0.25% *v*/*v* acetonitrile in water (1% *v*/*v* FA), then acetonitrile was increased to 50% over 20 min and maintained at 50% for 20 min with the flux at 1 mL/min. For Group C, elution started at 10% *v*/*v* acetonitrile in water (0.2% *v*/*v* TFA), then acetonitrile was increased as follows: to 10% at *t* = 5, 15% at *t* = 20, 15% at *t* = 25, 18% at *t* = 30, and 40% at *t* = 50 min, and maintained for 10 min with the flux at 0.5 mL/min.

## 3. Results

### 3.1. Structural Elucidation

**Figure 1:**
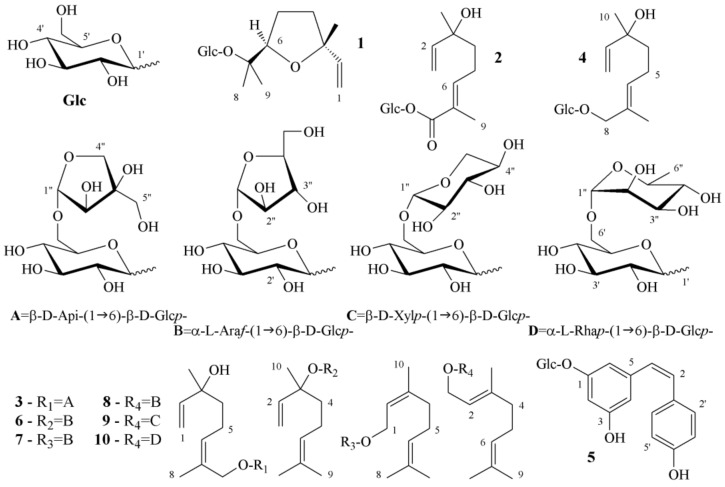
Structural formulae of previous (**1**–**3**) and present (**4**–**10**) flavor glycoconjugates isolated from Muscat cultivars. Arbitrary numbering.

The structures of compounds **4**–**10** were elucidated based on multiple-stage mass spectrometry (MS^n^) fragmentations (Table S1) and 1D- and 2D-NMR data (Figures S1–S9). Mostly, data matched that from the literature. The compounds **4**–**10** were isolated in small amounts and all had previously been described, so their ^13^C-NMR resonances were extracted from the HMBC correlation maps. Figure 1 shows the structural formulae of previous (**1**–**3**) and present (**4**–**10**) flavor glycoconjugates isolated from Muscat cultivars.

Compound **4** showed a molecular ion at *m*/*z* 355 [M + Na]^+^, which may correspond to the formula C_16_H_28_O_7_. The fragments observed in the MS^2^ experiment at *m*/*z* 203 [M − 152 + Na]^+^ and *m*/*z* 193 [M − 162 + Na]^+^ indicated the loss of a dehydrated monoterpene moiety (C_10_H_16_O) and a dehydrated hexose residue (C_6_H_10_O_5_), respectively. The proton spectrum exhibited signals for a vinyl group, one singlet methyl, one olefinic methyl group, an AB system for an oxymethylene group, and resonances that accounted for a β-glucoside. Detailed analysis of 2D-NMR spectra and comparison with literature revealed that compound **4** corresponded to betulalbuside A [9,10]. 

Compound **5** had a molecular ion of *m*/*z* 389 [M − H]ˉ, suggesting the formula C_20_H_22_O_8_. The MS^2^ experiment on *m*/*z* 389 gave the product ion at *m*/*z* 227 [M − 162 − H]ˉ, which stands for a highly unsaturated aglycone (C_14_H_11_O_3_) after loss of a dehydrated hexose sugar. The proton spectrum showed signals attributable to a *p*-di-substituted benzene, a *m*,*m′*-tri-substituted benzene, a *cis*-di-substituted ethylene, and resonances that accounted for a β-glucoside. 2D-NMR data matched that reported in the literature for (*Z*)-piceid [11].

The molecular ion of compound **6** was observed at *m*/*z* 471 [M + Na]^+^, which may account for the formula C_21_H_36_O_10_. In fact, the fragmentation experiments exhibited MS^2^ ion at *m*/*z* 333 (loss of dehydrated terpene plus oxidation) that gave rise to MS^3^ ions at *m*/*z* 201 (loss of dehydrated pentose) or *m*/*z* 155 (loss of gluconolactone). The proton spectrum exhibited signals, such as a vinyl group, a singlet methyl, and an olefinic proton (broad triplet) coupled to two allylic methyl groups, which fit the structure of linalool. In addition, there were resonances that accounted for a β-glucoside and an α-arabinofuranoside. Careful examination of 2D-NMR spectra and comparison with the literature established that Schievano et al. [7] have previously isolated compound **6**.

Compound **7** showed the molecular ion and MS^n^ fragments at *m*/*z* values identical to those of compound **6**. Compound **8** still had a molecular ion of *m*/*z* 471, as for compounds **6** and **7**. Moreover, the MS^2^ experiment furnished ions at *m*/*z*: 339 (loss of dehydrated pentose), 335 (loss of dehydrated terpene), and 333 (loss of dehydrated terpene plus oxidation). The ^1^H-NMR spectra of compounds **7** and **8** contained the same resonances of the disaccharide moiety that were observed for compound **6**. Consequently, the aglycones of compounds **6**, **7**, and **8** must be structural isomers. The terpene portion of **7** and **8** showed an olefinic proton (broad triplet) coupled to two allylic methyl groups and an oxymethylene group at a trisubstituted double bond. NOESY maps differentiated the aglycones of compounds **7** and **8**, enabling identification of neryl and geranyl structures, respectively. A search of the literature confirmed that Voirin et al. [12] had synthesized compound **7**, whereas compound **8** was isolated and characterized from rose flowers [13] and yellow Muscat [7]. 

MS measurements of compound **9** revealed *m*/*z* values identical to those of compound **8**. The 2D-NMR spectra confirmed the presence of the geranyl aglycone and of a β-glucoside. The resonances of the second sugar residue accounted for a α-xylopyranoside. Data obtained for compound **9** matched that for the geranyl primeveroside isolated from ginger rhizomes [14]. 

Compound 10 had a molecular ion of *m*/*z* 485 [M + Na]^+^, which was consistent with the formula C_22_H_38_O_10_. The MS^2^ fragments observed at *m*/*z* 349 [M + Na − 136]^+^ and 339 [M + Na − 146]^+^ indicated an aglycon moiety (C_10_H_17_O) linked to a rutinose sugar (C_12_H_21_O_9_) because of the typical loss of a dehydrated rhamnose (146 Da). The signals observed for its proton spectrum indicated the geranyl structure, thus compound **10** occurs in “Moscato Giallo” grapes [7].

### 3.2. Comparison of RP- and HILIC-HPLC Chromatograms

A comparison of chromatographic performances of RP- and HILIC-HPLC was performed at the analytical scale. Thus, the “Moscato Rosa” sample and each pure compound were injected into analytical RP and HILIC columns. In order to provide a larger data set, compounds previously isolated from “Moscato Giallo” by preparative HILIC-HPLC were also analyzed [8]. The most reliable detector was found to be the ELSD, because GAPs showed faint UV absorption. The ELSD showed high sensitivity, provided a universal response, and gave a fairly uniform mass response.

**Figure 2:**
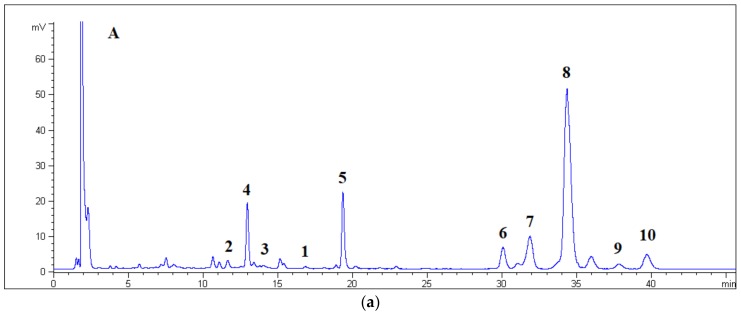

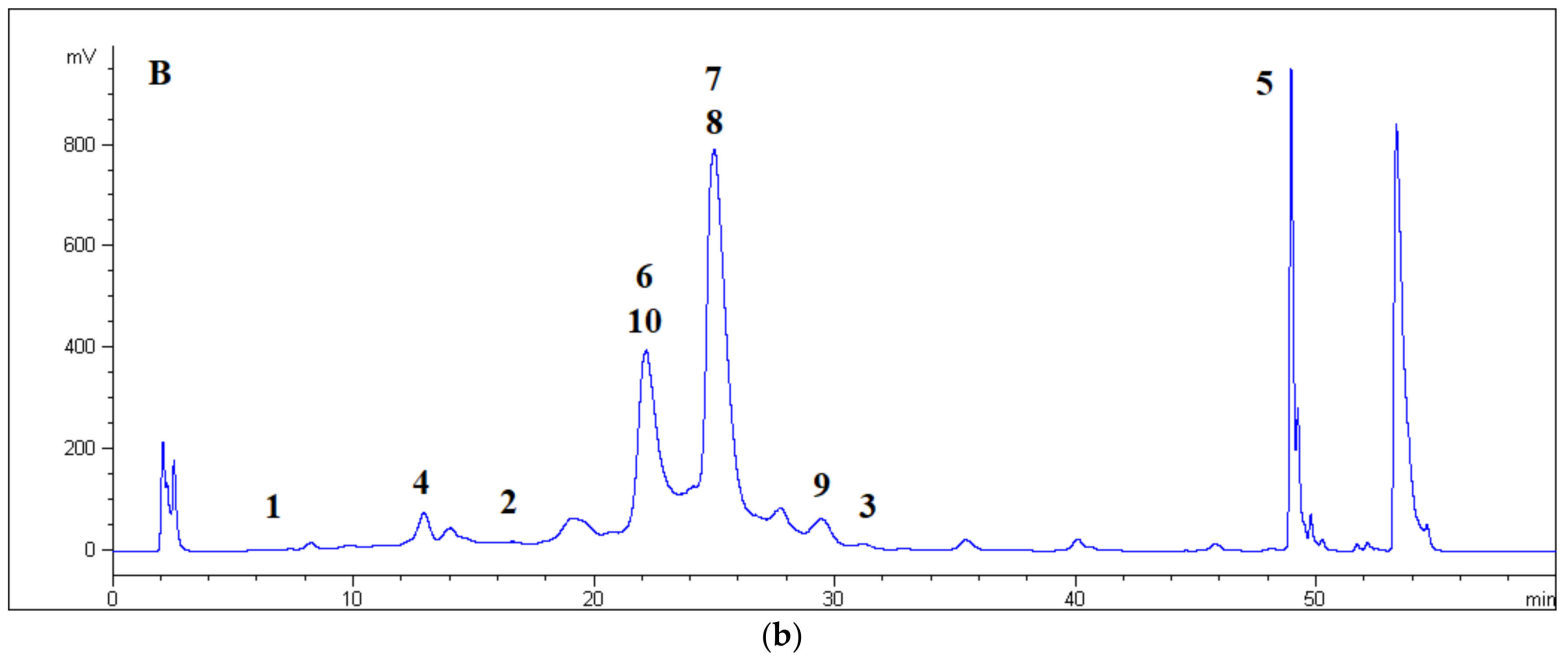
HPLC chromatograms of the GAP fraction obtained from “Moscato Rosa” (compounds **4**–**10**). The figure also shows, close to their *t_R_*, the compounds **1**–**3**, which were previously isolated from “Moscato Giallo” [8] and injected separately. Panels: (**a**) RP column, (**b**) HILIC column.

The RP- and HILIC-HPLC chromatograms of the “Moscato Rosa” sample are shown in Figure 2. The retention times (*t_R_*) of compounds are summarized in Table 1.

### 3.3. Effectiveness of Pb(II) Treatment 

Experimentally, treatment of the grape juice extract with lead acetate was found to be the determinant step of sample preparation. The formation of insoluble lead complexes of individual phenolic compounds was therefore investigated using an HPLC-UV method. Three classes of compounds—simple phenols, flavonoids, and anthocyanins—were subjected to lead acetate treatment at two pH levels and three molar equivalent ratios. After precipitation, the solutions were centrifuged and the resulting supernatants injected into an RP-HPLC column detecting at λ = 280 nm. The integrated area of each chromatographic peak in treated samples was measured and compared to peaks from the starting (untreated) sample. Table 2 reports the percentage of precipitation for each compound. 

## 4. Discussion

Aim 1. In this paper, the structural formula of seven GAPs were characterized: three (**5**, **6**, and **8**) were previously identified in grapes, two (**7**, **10**) were obtained by synthesis, whereas **4** was isolated from *Viburnum orientale* and **9** from *Zingiber officinale*. Herein, we report four compounds (**4**, **7**, **9**, and **10**) for the first time in a grape juice. Geraniol was found to be linked to several disaccharides (**8**, **9**, and **10**) whereas arabinose occurs as furanoside (**6**, **7**, and **8**) rather than the common vicianoside. Notably, the primeverose and rutinose sugars were also present. Finally, (*E*)-hydroxylinalool (**4**) was identified in “Moscato Rosa”, whilst the (*Z*) isomer (**3**) occurred in “Moscato Giallo”.

Aim 2. The separation of GAP is a consequence of the type and the number of hydrophilic functional groups (HFGs) that can form hydrogen bonds. Few HFGs in the aglycone moiety will increase the *t_R_* during RP elution, whereas many HFGs in the carbohydrate moiety will increase the *t_R_* during normal phase HILIC elution. In fact, the sugar portion is poorly soluble in the hydrophobic layer of the bonded ODS particles and, vice versa, the aglycone portion is poorly soluble in the aqueous layer covering the bonded ZIC particles. The chemical structure of (*Z*)-piceid (**5**) was different so its *t_R_* was not comparable to those of GAPs (**1**–**4**, **6**–**10**).

The order of elution in the RP mode can be rationalized by examining the chemical structure of the aglycone moiety. With respect to the geraniol molecule (**8**, **9**, and **10**), compounds **2**, **3**, and **4** had an additional hydroxyl group. These three compounds were first eluted, irrespective of their number of monosaccharide units. In fact, **3** (which comprised two sugar units) showed a higher *t_R_* than **4** (which only comprised one sugar unit). The discriminating factor between **3** and **4** was therefore the configuration at the double bond of the hydroxylinalool, which was found to be Z and E, respectively. With respect to the geraniol molecule, compound **1** possessed an ether functionality, which acts only as acceptor of hydrogen bonds such that **1** eluted at an intermediate *t_R_*. Below were the compounds **6**, **7**, and **8**, which had identical disaccharide moieties, whereas their terpenoid portion comprised linalool, nerol, and geraniol, respectively. Again, it was possible to observe discrimination between geometric isomers. The last eluted compounds were two glycosylated geraniol molecules, which were characterized by the presence of xylopyranose (**9**) and rhamnose (**10**) units. The xylopyranose sugar possessed one less hydroxyl group than the arabinofuranose; the rhamnose unit had an apolar methyl group.

The normal-phase HILIC allowed partitioning of the glycosidic moieties, differentiating monosaccharides and disaccharides. The compounds that were glycosylated by one sugar unit were eluted first because they were less soluble in the aqueous layer covering the stationary phase. Moreover, compounds **1**, **4**, and **2** showed the reverse order of elution with respect to the ODS stationary phase. The hydroxylinalool disaccharide of **3** became the last eluted aroma precursor by HILIC. Focusing on the set of five disaccharides linked to hydrophobic monoterpenes (i.e., **6**–**10**), the least retained compound appeared to again be compound **6**. The next eluted compound was **10**, with a rhamnose unit. Compounds **7** and **8** had identical disaccharide linkages and eluted together because they differed only in their configuration at the double bond of the terpene.

Aim 3. The use of lead (II) acetate for several purposes has been reported in recent literature [15] and is considered an official method of analysis [16]. However, a detailed mechanism of action has not yet been described. Two articles concerning the purification of anthocyanins from cranberry juice [17] or wine [18] using lead (II) acetate reported the quantity of precipitated anthocyanins, determined either by a pH differential [17] or a spectrophotometric method [18]. In summary, the literature stated that: (i) compounds possessing an *o*-dihydroxy benzene moiety are easily precipitated, while simple phenols form lead salts to a low extent; and (ii) basic lead acetate [Pb(OAc)_2_·2Pb(OH)_2_] is more effective than neutral lead acetate [Pb(OAc)_2_].

It is evident from Table 2 the importance of an *o*-dihydroxy benzene moiety. In fact, caffeic acid forms the insoluble lead salt exhaustively even at acidic pH and at half-molar equivalence. In comparison, the complete precipitation of coumaric and ferulic acids can only be achieved at basic pH and with two molar equivalents. The carboxylic functionality of cinnamic acid acts to a certain extent because cinnamic acid does not possess a phenol group. The presence of an *m*-dihydroxy benzene moiety is ineffective, so most of the stilbenoid resveratrol remained dissolved. The flavanol naringin and the flavone diosmin formed insoluble salts partially because they lacked the *o*-dihydroxy benzene group. Interestingly, the flavonol kaempferol was easily precipitated, suggesting that the hydroxyl at the C-3 position played somewhat of a role. This was confirmed by the decreased precipitation of kaempferol 3-*O*-glucoside. The flavone luteolin 7-*O*-glucoside and the flavonol quercetin formed insoluble complexes at acidic pH and at half-molar equivalence. Finally, the anthocyans cyanidin and mirtillin could be precipitated at an acidic pH, whereas malvin could only be precipitated at a basic pH. In conclusion, data confirmed our previous statements in the literature and underlined that the effectiveness of precipitation is derived from the structural features of individual phenolic compounds. In addition, a larger extent of precipitation might be achieved by using excess lead acetate rather than basic pH conditions; this avails also because the content of phenolic compounds in crude natural extracts is difficult to establish. The lead acetate procedure enables uninteresting phenolic compounds to be discarded, but they cannot be easily recovered after precipitation. 

Preliminary experiments suggest polyvinylpolypyrrolidone (PVPP) has different binding properties compared to lead (II) acetate.

## 5. Conclusions

This study is the first on grapes from the “Moscato Rosa” cultivar. We have previously demonstrated that treatment with lead (II) acetate effectively removes phenols from the solid phase extract of grape juice and enables the purification of GAPs. The chromatographic separation of GAPs can be achieved by both HILIC- and RP-HPLC stationary phases. Moreover, the two methods offer distinct separation performance and can be used in sequence to improve the results of future studies. The characterization of GAPs by NMR spectroscopy is laborious but this technique provides unambiguous structural identification. We report, for the first time, the isolation of compounds **4** and **9** from grape juice, and compound **7** from a natural source. In addition, identification of the primeverose saccharide of **9** is novel for grape juice. Finally, our procedure for studying aroma precursors is also applicable to wine and other juices or plant extracts. It could be further improved to implement quantitative determination of GAPs.

## Figures and Tables

**Table 1:** Retention times (*t_R_*) on analytical RP and hydrophilic interaction liquid chromatography (HILIC) columns of the glycosidic aroma precursors 1–10 and their hydrophilic functional groups (HFG) or total number of hydroxyls.

Reversed Phase			HILIC		
*t_R_* (min)	Compound	HFG	*t_R_* (min)	Compound	Total-OH
11.6	2	C = O + C-OH	6.7	1	4
12.9	4	C-OH	12.8	4	5
14.0	3	C-OH	16.4	2	5
16.8	1	C-O-C	21.9	6	6
19.3	5	C-OH	22.0	10	6
30.0	6	-	25.0	7	6
31.7	7	-	25.0	8	6
34.1	8	-	29.4	9	6
37.8	9	-	31.1	3	7
39.6	10	-	48.9	5	6

**Table 2:** Percentage of precipitated phenol after treatment with lead (II) acetate at the specified pH.

Entry	Compound	pH	0.5 ^1^	1.0 ^1^	2.0 ^1^
1	Cinnamic acid	~5	26	30	30
		~8	17	36	36
2	Coumaric acid	~5	23	33	34
		~8	24	43	100
3	Caffeic acid	~5	98	100	100
		~8	99	100	100
4	Ferulic acid	~5	44	66	68
		~8	61	83	100
5	Resveratrol	~5	6	6	11
		~8	13	20	35
6	Naringin	~5	16	25	19
		~8	23	29	33
7	Diosmin	~5	-	32	54
		~8	-	12	49
8	Kaempferol	~5	74	79	88
		~8	100	100	100
9	Kaempferol 3-*O*-glucoside	~8	40	100	100
10	Luteolin 7-*O*-glucoside	~5	100	100	100
		~8	100	100	100
11	Quercetin	~5	100	100	100
		~8	100	100	100
12	Cyanidin	~5	45	81	100
		~8	100	100	100
13	Mirtillin	~5	93	93	93
		~8	100	100	100
14	Malvin	~5	37	27	46
		~8	100	100	100

^1^ Molar equivalents of lead (II) acetate added to each aliquot of phenolic compounds.

## Supplementary Materials

The following are available online at http://www.mdpi.com/2304-8158/8/1/28/s1, Figure S1: The ^1^H-NMR spectrum of **4**, Figure S2: The ^1^H-NMR spectrum of **5** (CD_3_OD), Figure S3: The ^1^H-NMR spectrum of **5** (D_2_O), Figure S4: The ^1^H-NMR spectrum of **6**, Figure S5: The ^1^H-NMR spectrum of **7**, Figure S6: The ^1^H-NMR spectrum of **8**, Figure S7: The ^1^H-NMR spectrum of **9**, Figure S8: The ^1^H-NMR spectrum of **10**, Figure S9: The 1D- and 2D-NMR data of compounds **4**–**10**, Table S1: Results of fragmentation experiments (MS^2^) for compounds **4**–**10**.
