# Supplementary material for: Advances in the Dereplication of Aroma Precursors from Grape Juice by Pretreatment with Lead Acetate and Combined HILIC- and RP-HPLC Methods
Source: Foods. 2019 Jan 15;8(1):28. doi: 10.3390/foods8010028 (PMC6351915; doi:10.3390/foods8010028)
Supplement: Supplementary file 1 [file foods-08-00028-s001.pdf]

Vitis vinifera L. cultivar 'Moscato Rosa'

Advances in the Dereplication of Aroma Precursors from Grape Juice by Pretreatment with Lead Acetate and Combined Hilic- and RP-Hplc Methods

Michele D'Ambrosio

Laboratory of Bioorganic Chemistry, Department of Physics, Università degli Studi di Trento, 38123 Trento, Italy

Correspondence should be addressed to: Tel: +39-0461-281509. Fax: +39-0461-281696. *E-mail address*: michele.dambrosio@unitn.it

Table. S1. Results of fragmentation experiments (MS^2) for compounds **4 - 10**

Compound			Molecular Ion Mass[1]	Fragment		Neutral Loss		
Num.	Mass	Formula		Mass	Formula	Mass	Formula	Description
4	332	$C_{16}H_{28}O_7$	355	203	$C_6H_{12}O_6$	152	$C_{10}H_{16}O$	Dehydrated Terpene
				193	$C_{10}H_{18}O_2$	162	$C_6H_{10}O_5$	Dehydrated Hexose
5	390	$C_{20}H_{22}O_8$	389	227	$C_{14}H_{11}O_3$	162	$C_6H_{10}O_5$	Dehydrated Hexose
6 – 7	448	$C_{21}H_{36}O_{10}$	471	333	$C_{11}H_{18}O_{10}$	138	$C_{10}H_{16}+H_2$	Dehydrated Terpene + Oxidation
				201	$C_6H_{10}O_6$	*132*	$C_5H_8O_4$	*Dehydrated Pentose*
				155	$C_5H_8O_4$	*178*	$C_6H_{10}O_6$	*Gluconolactone*
8 – 9	448	$C_{21}H_{36}O_{10}$	471	339	$C_{16}H_{28}O_6$	132	$C_5H_8O_4$	Dehydrated Pentose
				335	$C_{11}H_{20}O_{10}$	136	$C_{10}H_{16}$	Dehydrated Terpene
				333	$C_{11}H_{18}O_{10}$	138	$C_{10}H_{16}+H_2$	Dehydrated Terpene + Oxidation
10	462	$C_{22}H_{38}O_{10}$	485	349	$C_{12}H_{22}O_{10}$	136	$C_{10}H_{16}$	Dehydrated Terpene
				339	$C_{12}H_{21}O_9$	146	$C_6H_{10}O_4$	Dehydrated Rhamnose

[1] The formula of molecular ions is $[M+Na]^+$ but for compound **5** is $[M-H]^-$. Lines in italics are from MS^3 experiments

Fig. S1. The ^{1}H-NMR spectrum of **4**

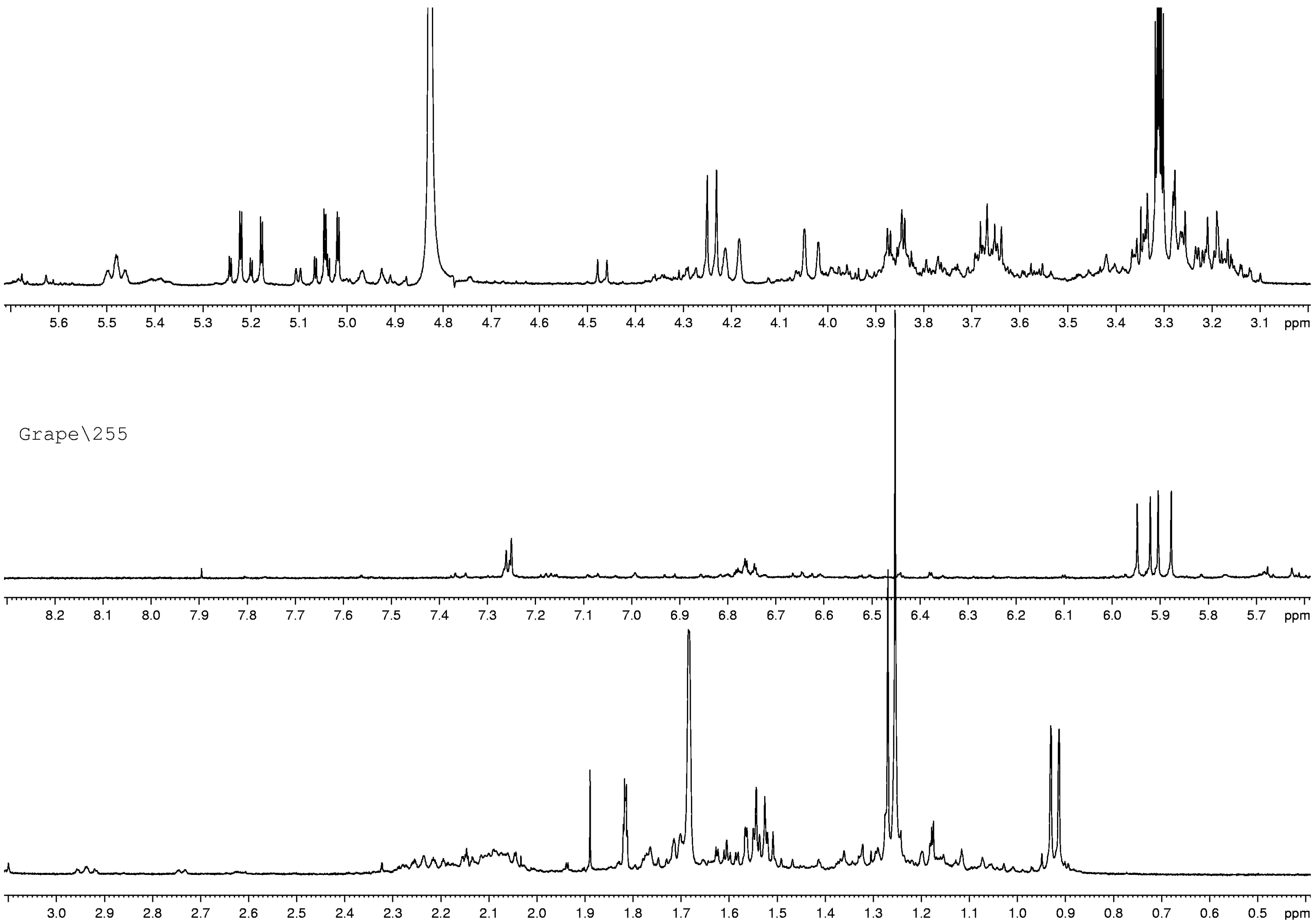

Fig. S2. The ^{1}H-NMR spectrum of **5** (CD_3OD)

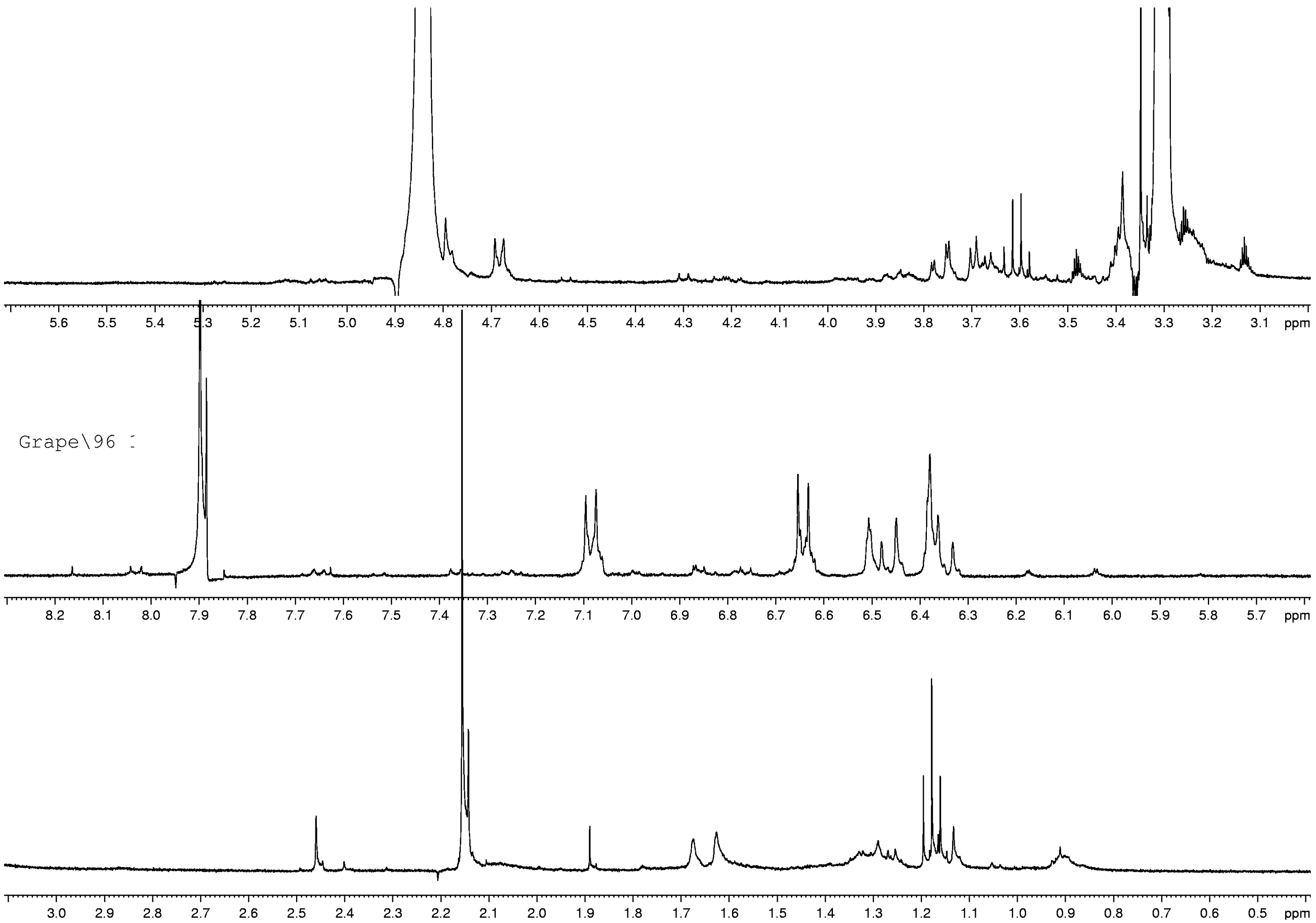

Fig. S3. The ^{1}H-NMR spectrum of **5** (D_2O)

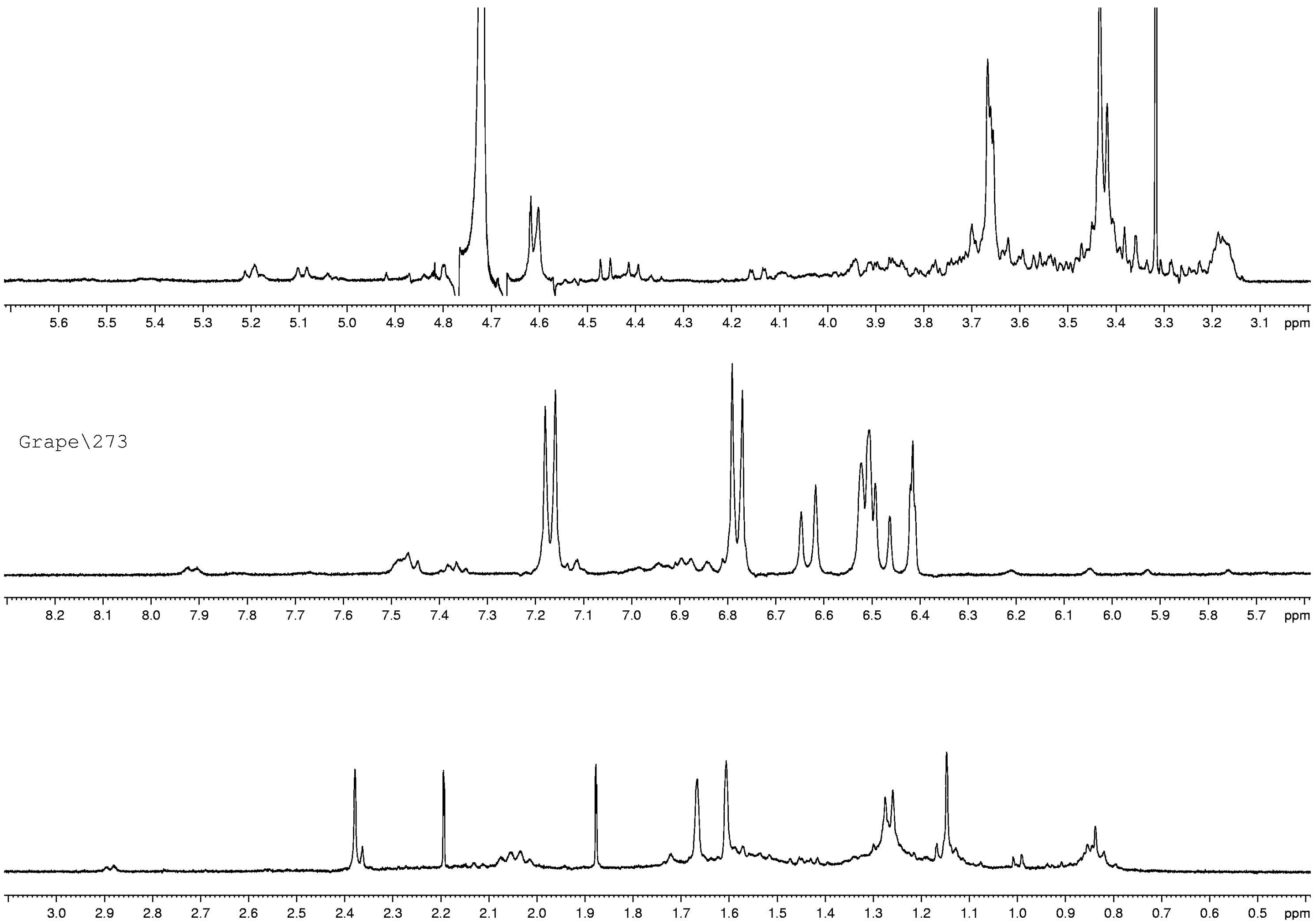

Fig. S4. The ^{1}H-NMR spectrum of **6**

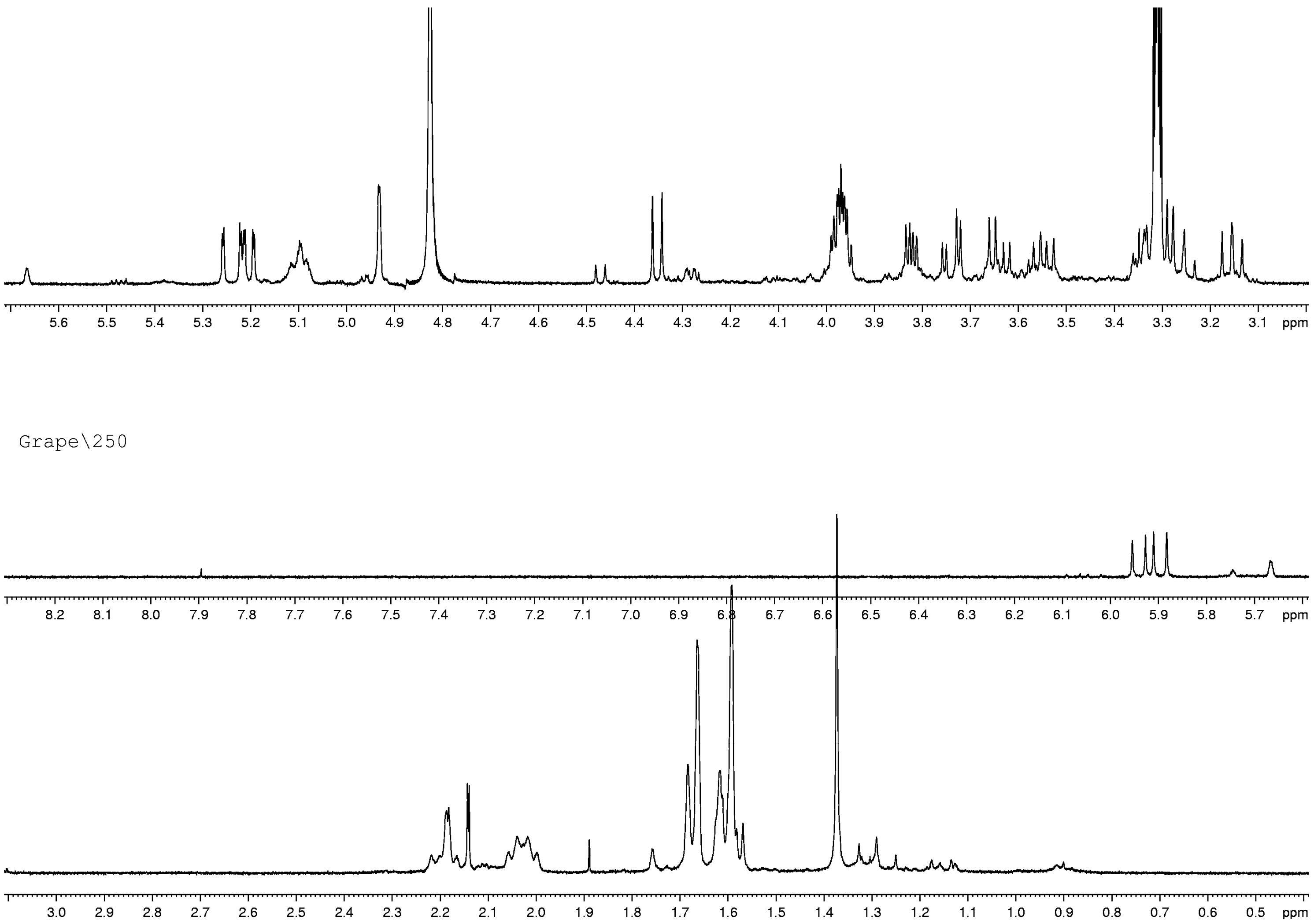

Fig. S5. The ^{1}H-NMR spectrum of **7**

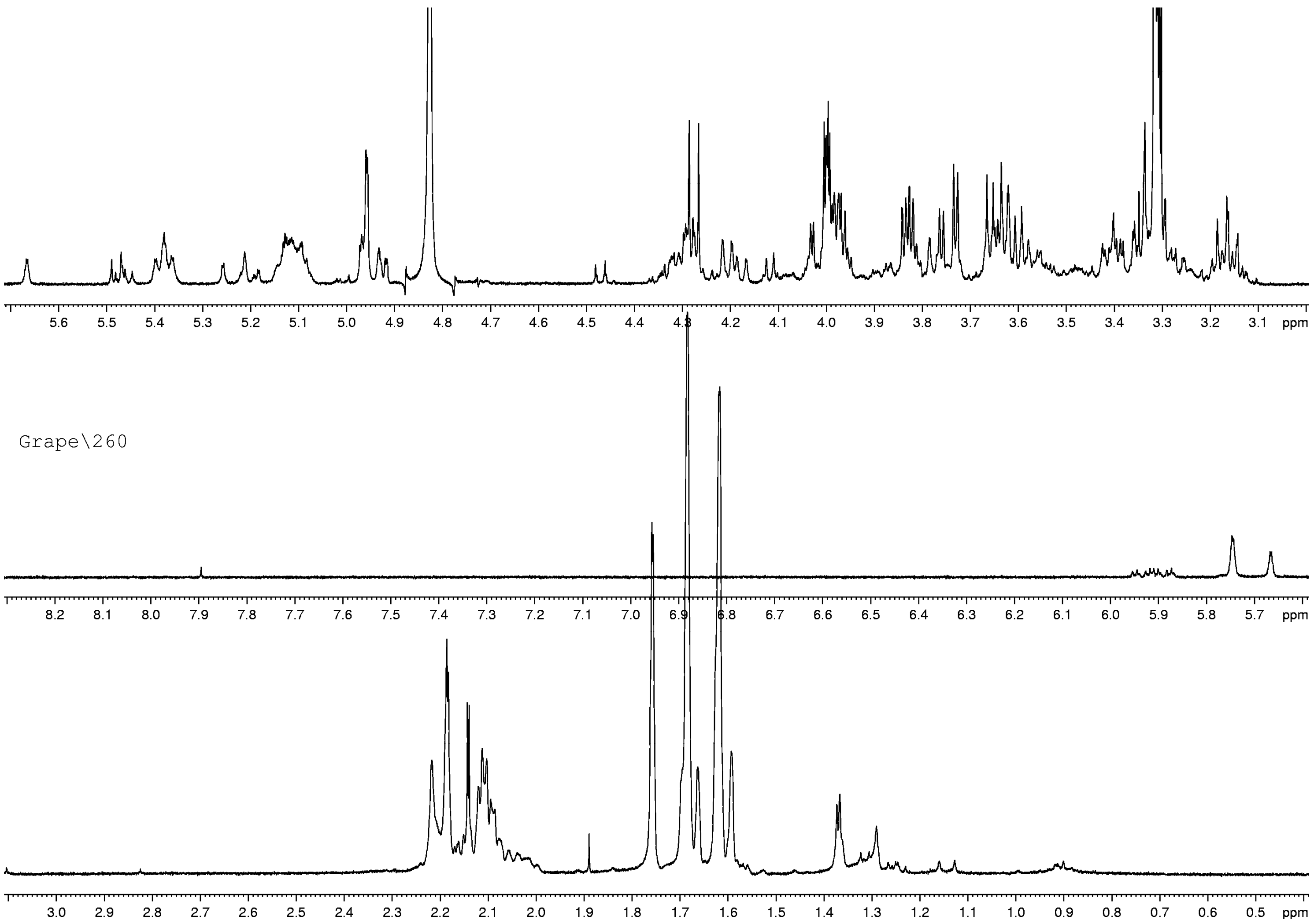

Fig. S6. The ^{1}H-NMR spectrum of **8**

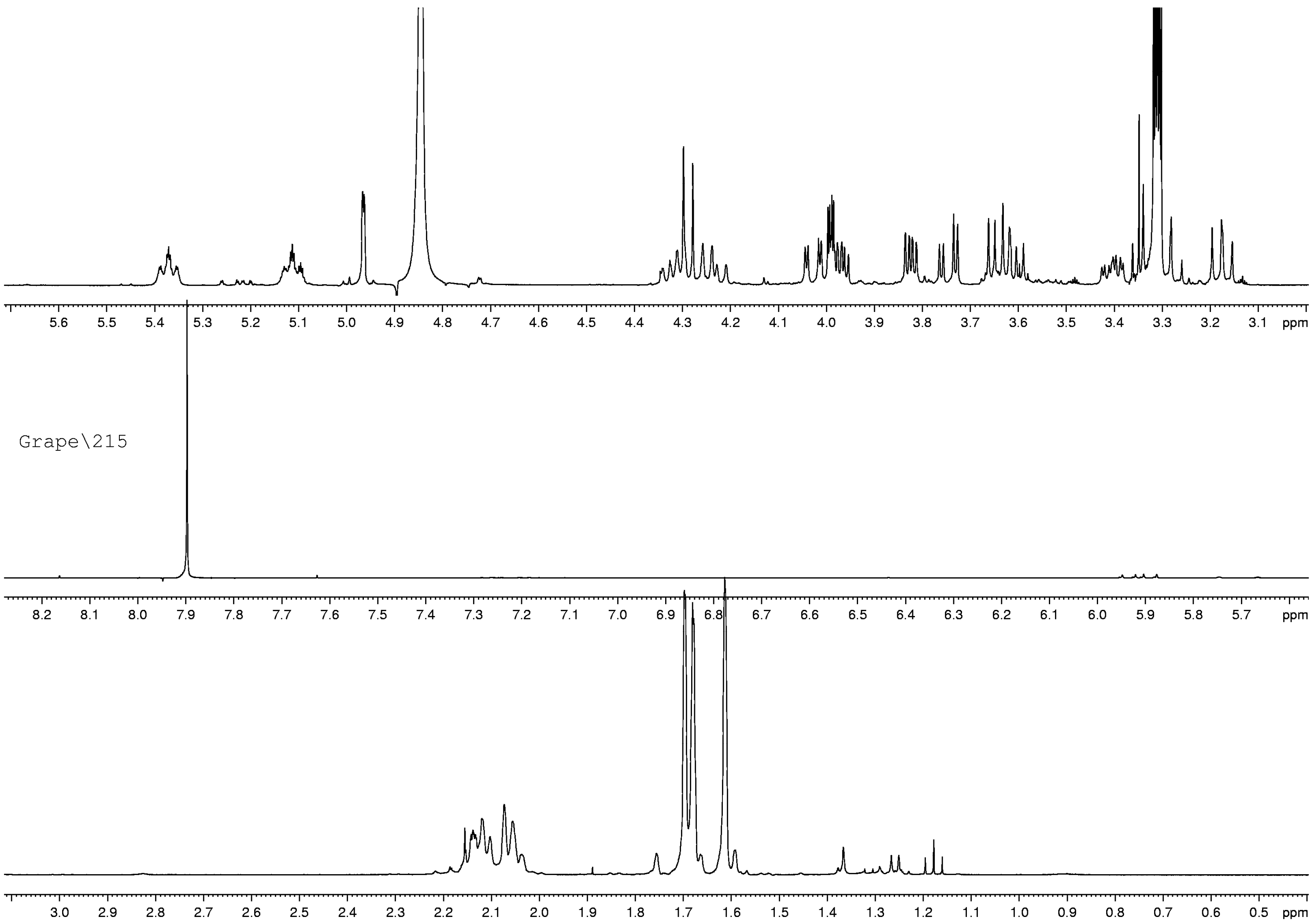

Fig. S7. The ^{1}H-NMR spectrum of **9**

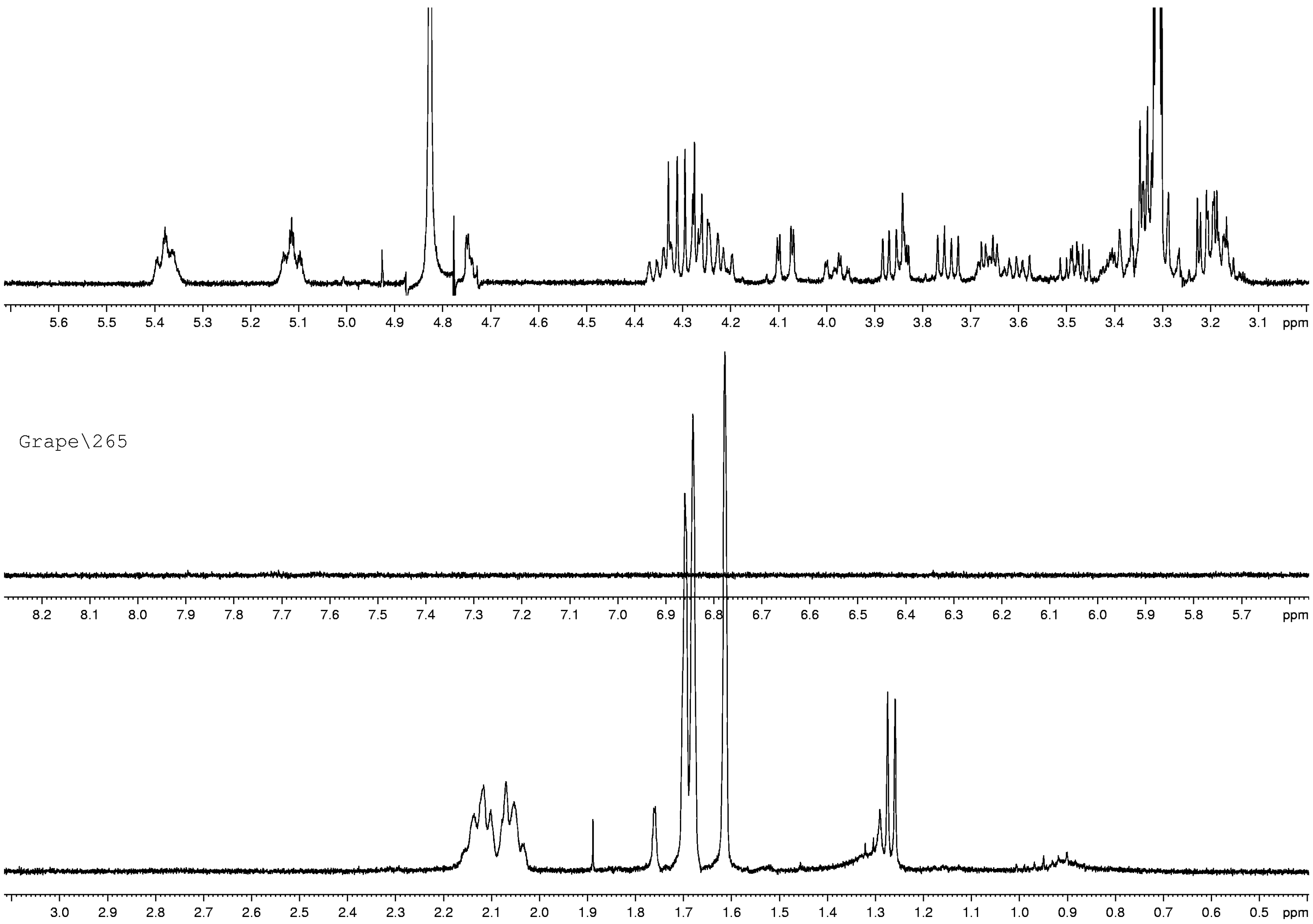

Fig. S8. The ^{1}H-NMR spectrum of **10**

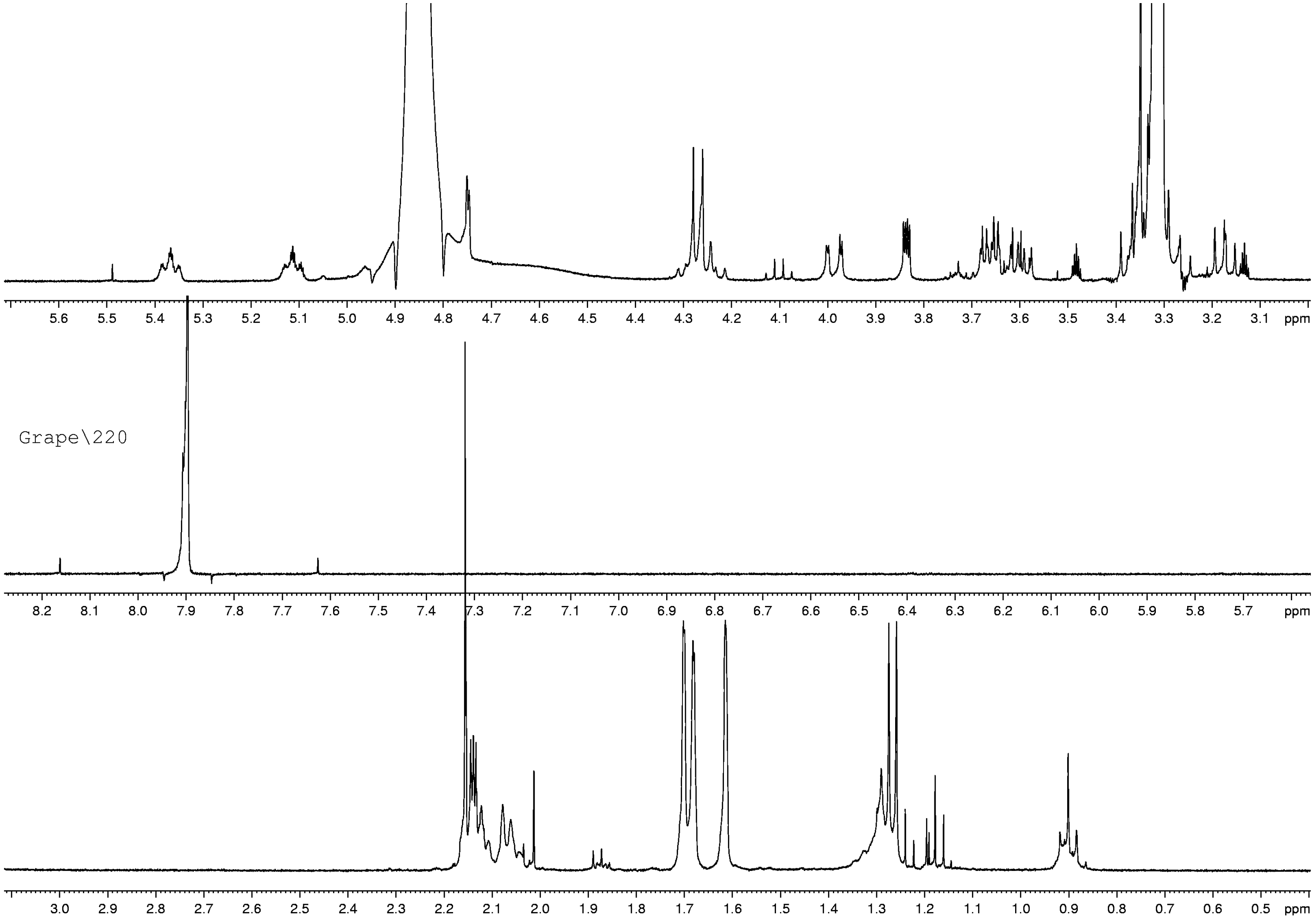

Fig. S9. The 1D- and 2D-NMR data of compounds **4-10**

(2*E*,6*R)-6-hydroxy-2,6-dimethyl-2,7-octadien-1-yl β-D-glucopyranoside (4)**

^{1}H-NMR (CD_3OD, 400 MHz): δ 5.91 (1H, dd, *J* = 17.3, 10.7 Hz, H-2), 5.48 (1H, br.t, *J* = 7.6 Hz, H-6), 5.20 (1H, dd, *J* = 17.3, 1.6 Hz, Ht-1), 5.03 (1H, dd, *J* = 10.7, 1.6 Hz, Hc-1), 4.24 (1H, d, *J* = 8.0 Hz, H-1'), 4.20 and 4.04 (2H, AB, *J* = 11.3 Hz, H-8), 3.86 (1H, dd, *J* = 11.9, 2.4 Hz, Ha-6'), 3.66 (1H, dd, *J* = 11.9, 5.7 Hz, Hb-6'), ~3.42 (1H, m, H-5'), ~3.34 (1H, m, H-3'), ~3.28 (1H, m, H-4'), ~3.16 (1H, m, H-2'), ~2.09 (2H, m, H-5), 1.68 (3H, br.s, H-9), ~1.54 (2H, m, H-4), 1.26 (3H, s, H-10); ^{13}C-NMR (CD_3OD, 100 MHz): δ 145.9 (C-2), 132.6 (C-7), 129.9 (C-6), 111.8 (C-1), 102.4 (C-1'), 77.8 (C-3'), 77.5 (C-5'), 74.8 (C-2'), 73.4 (C-3), 71.4 (C-4'), 62.4 (C-6'), 75.6 (C-8), 42.5 (C-4), 27.7 (C-10), 23.3 (C-5), 13.7 (C-9). NOESY (CD_3OD, 400 MHz): 2H-5 ↔ 3H-9; H-6 ↔ 2H-8; 3H-10 ↔ H-2 and Hc-1. HMBC (CD_3OD, 400 MHz): C8 → 3H-9 and H-1'; C7 → 2H-8 and 3H-9; C6 → 2H-8 and 3H-9; C3 → H-2 and 2H-1 and 3H-10; C1' → 2H-8.

3-hydroxy-5-[(1*Z*)-2-(4-hydroxyphenyl)ethenyl]phenyl β-D-glucopyranoside (5)

^{1}H-NMR (D_2O, 400 MHz): δ 7.17 (2H, d, *J* = 8.5 Hz, H-3' and H-5'), 6.78 (2H, d, *J* = 8.5 Hz, H-2' and H-6'), 6.63 (1H, d, *J* = 12.0 Hz, H-8), 6.52 (1H, br.s, H-6), 6.51 (1H, br.s, H-2), 6.48 (1H, d, *J* = 12.0 Hz, H-7), 6.41 (1H, br.s, H-4), 4.61 (1H, d, *J* = 7.0 Hz, H-1'), ~3.67 (2H, m, H-6'), ~3.43 (3H, m, H-2' and H-3' and H-4'), ~3.18 (1H, m, H-5'); ^{13}C-NMR (D_2O, 100 MHz): δ 158.4 (C-1), 141.2 (C-1), 132.0 (C-8), 129.2 (C-7), 112.1 (C-6), 108.7 (C-2), 104.4 (C-4), 156.3 (C-4'), 131.5 (C-2' and C-6'), 130.8 (C-1'), 116.4 (C-3' and C-5'), 101.5 (C-1''), 77.0 (C-5''), 76.7 (C-3''), 73.7 (C-2''), 70.0 (C-4''), 61.3 (C-6''). NOESY (D_2O, 400 MHz): H-1'' ↔ H-2 and H-4. HMBC (D_2O, 400 MHz): C1 → H-8; C3 → H-2 and H-1''; C4' → H-2'; C1' → H-7 and H-3'.

1-ethenyl-1,5-dimethyl-4-hexen-1-yl α-L-arabinofuranosyl-(1→6)-β-D-glucopyranoside (6)

^{1}H-NMR (CD_3OD, 400 MHz): δ 5.92 (1H, dd, *J* = 17.7, 10.9 Hz, H-2), 5.24 (1H, dd, *J* = 17.7, 1.2 Hz, Ht-1), 5.21 (1H, dd, *J* = 10.9, 1.2 Hz, Hc-1), 5.10 (1H, br.t, *J* = 7.6 Hz, H-6), 4.93 (1H, d, *J* = 1.0 Hz, H-1''), 4.35 (1H, d, *J* = 7.8 Hz, H-1'), 3.98 (1H, m, *sup*, H-2''), ~3.97 (1H, m, *sup*, Ha-6'), 3.96 (1H, m, *sup*, H-4''), 3.82 (H, dd, *J* = 5.8, 3.1 Hz, H-3''), 3.74 (1H, dd, *J* = 11.8, 3.3 Hz, Ha-5''), 3.64 (1H, dd, *J* = 11.8, 5.3 Hz, Hb-5''), 3.55 (1H, dd, *J* = 10.9, 5.9 Hz, Hb-6'), 3.33 (1H, m, *sup*, H-5'), ~3.31 (1H, m, *sup*, H-3'), ~3.28 (1H, m, *sup*, H-4'), 3.15 (1H, dd, *J* = 8.8, 8.0 Hz, H-2'), ~2.03 (2H, m, H-5), 1.66 (3H, br.s, H-8), ~1.60 (2H, m, H-4), 1.59 (3H, br.s, H-9), 1.37 (3H, s, H-10); ^{13}C-NMR (CD_3OD, 100 MHz): δ 144.0 (C-2), 132.0 (C-7), 125.3 (C-6), 115.4 (C-1), 109.5 (C-1''), 99.2 (C-1'), 85.6 (C-4''), 82.9 (C-2''), 78.7 (C-3''), 77.8 (C-3'), 76.1 (C-5'), 74.8 (C-2'), 71.7 (C-4'), 67.8 (C-6'), 81.2 (C-3), 62.7 (C-5''), 42.2 (C-4), 24.1 (C-5), 25.6 (C-8), 22.9 (C-10), 17.4 (C-9). NOESY (CD_3OD, 400 MHz): H-6 ↔ 3H-8; H-1' ↔ 3H-10 and H-2; H-1'' ↔ Hb-6'. HMBC (CD_3OD, 400 MHz): C3 → Ht-1 and 3H-10 and H-1'; C2 → 3H-10; C4 → 3H-10; C6' → H-1''; C5'' → H-1''.

(2*Z*)- 3,7-dimethyl-2,6-octadien-1-yl α-L-arabinofuranosyl-(1→6)-β-D-glucopyranoside (7)

^{1}H-NMR (CD_3OD, 400 MHz): δ 5.38 (1H, br.t, H-2), 5.13 (1H, br.t, H-6), 4.96 (1H, d, *J* = 1.4 Hz, H-1''), 4.28 (1H, d, *J* = 7.8 Hz, H-1'), ~4.30 (1H, dd, *J* = 17.3, 1.6 Hz, Ha-1), 4.19 (1H, dd, *J* = 10.7, 1.6 Hz, Hb-1), 4.01 (1H, dd, *J* = 11.0, 2.4 Hz, Ha-6'), 3.99 (1H, dd, *J* = 3.2, 1.4 Hz, H-2''), 3.97 (1H, ddd, *J* = 5.8, 5.3, 3.3 Hz, H-4''), 3.83 (H, dd, *J* = 5.8, 3.2 Hz, H-3''), 3.74 (1H, dd, *J* = 12.0, 3.3 Hz, Hb-5''), 3.64 (1H, dd, *J* = 12.0, 5.3 Hz, Hb-5''), 3.62 (1H, dd, *J* = 11.0, 5.6 Hz, Hb-6'), 3.41 (1H, ddd, *J* = 9.4, 5.6, 2.4 Hz, H-5'), ~3.34 (1H, m, *sup*, H-3'), ~3.28 (1H, m, *sup*, H-4'), 3.16 (1H, dd, *J* = 9.0, 7.8 Hz, H-2'), ~2.10 (4H, m, H-4 and H-5), 1.76 (3H, br.s, H-10), 1.69 (3H, br.s, H-8), 1.62 (3H, br.s, H-9); ^{13}C-NMR (CD_3OD, 100 MHz, $δ_C$ extracted from HMBC): δ 141.6 (C-3), 132.6 (C-7), 124.7 (C-6), 122.2 (C-2), 109.6 (C-1''),102.6 (C-1'), 85.6 (C-4''), 82.7 (C-2''), 78.6 (C-3''), 77.6 (C-3'), 76.2 (C-5'), 74.7 (C-2'), 71.6 (C-4'), 67.7 (C-6'), 66.2 (C-1), 62.6 (C-5''), 32.5 (C-4), 27.4 (C-5), 25.5 (C-8), 23.4 (C-10), 17.4 (C-9). NOESY (CD_3OD, 400 MHz): H-2 ↔ 3H-10; H-6 ↔ 3H-8. HMBC (CD_3OD, 400 MHz): C1 → H-1'; C3 → 2H-1 and 3H-10; C4 → 3H-10; C7 → 3H-8 and 3H-9; C-1' → 2H-1; C6' → H-1''; C5'' → H-1''.

(2*E*)- 3,7-dimethyl-2,6-octadien-1-yl α-L-arabinofuranosyl-(1→6)-β-D-glucopyranoside (8)

^{1}H-NMR (CD_3OD, 400 MHz): δ 5.37 (1H, br.t, H-2), 5.11 (1H, br.t, H-6), 4.96 (1H, d, *J* = 1.4 Hz, H-1''), 4.29 (1H, d, *J* = 7.8 Hz, H-1'), 4.32 (1H, dd, *J* = 11.8, 6.4 Hz, Ha-1), 4.23 (1H, dd, *J* = 11.8, 7.6 Hz, Hb-1), 4.03 (1H, dd, *J* = 11.1, 2.4 Hz, Ha-6'), 3.99 (1H, dd, *J* = 3.3, 1.4 Hz, H-2''), 3.97 (1H, ddd, *J* = 6.0, 5.4, 3.3 Hz, H-4''), 3.82 (2H, dd, *J* = 6.0, 3.3 Hz, H-3''), 3.74 (1H, dd, *J* = 11.9, 3.3 Hz, Ha-5''), 3.64 (1H, dd, *J* = 11.9, 5.4 Hz, Hb-5''), 3.62 (1H, dd, *J* = 11.1, 6.0 Hz, Hb-6'), 3.40 (1H, ddd, *J* = 9.4, 6.0, 2.4 Hz, H-5'), 3.34 (1H, t, *J* = 8.8 Hz, H-3'), 3.28 (1H, m, *J* = 9.4, 8.8 Hz, H-4'), 3.16 (1H, dd, *J* = 8.8, 7.8 Hz, H-2'), ~2.12 (2H, m, H-5), ~2.06 (2H, m, H-4), 1.70 (3H, br.s, H-10), 1.68 (3H, br.s, H-8), 1.61 (3H, br.s, H-9); ^{13}C-NMR (CD_3OD, 100 MHz): δ 141.9 (C-3), 132.1 (C-7), 124.8 (C-6), 121.0 (C-2), 109.6 (C-1''),102.4 (C-1'), 85.6 (C-4''), 82.9 (C-2''), 78.6 (C-3''), 77.7 (C-3'), 76.4 (C-5'), 74.7 (C-2'), 71.6 (C-4'), 67.7 (C-6'), 66.0 (C-1), 62.8 (C-5''), 40.5 (C-4), 27.1 (C-5), 25.6 (C-8), 17.3 (C-9), 16.2 (C-10). NOESY (CD_3OD, 400 MHz): H-2 ↔ 2H-4; H-6 ↔ 3H-8. HMBC (CD_3OD, 400 MHz): C1 → H-1'; C3 → 2H-1 and 3H-10; C4 → 3H-10; C7 → 3H-8 and 3H-9; C-1' → 2H-1; C6' → H-1''; C-1'' → 2H-6'; C5'' → H-1''.

(2*E*)- 3,7-dimethyl-2,6-octadien-1-yl β-D-xylopyranosyl-(1→6)-β-D-glucopyranoside (9)

^{1}H-NMR (D_2O, 400 MHz): δ 5.35 (1H, br.t, H-2), 5.17 (1H, br.t, H-6), 4.43 (1H, d, *J* = 8.0 Hz, H-1''), 4.42 (1H, d, *J* = 7.8 Hz, H-1'), ~4.30 (2H, m, H-1), 4.11 (1H, dd, *J* = 11.5, 2.0 Hz, Ha-6'), 3.93 (1H, dd, *J* = 11.7, 5.4 Hz, Ha-5''), 3.87 (1H, dd, *J* = 11.5, 5.3 Hz, Hb-6'), 3.59 (1H, ddd, *J* = 10.0, 9.0, 5.4, Hz, H-4''), 3.44 (1H, m, *sup*, H-5'), 3.41 (1H, t, *J* = 9.0 Hz, H-3'), 3.40 (1H, t, *J* = 9.0 Hz, H-4'), 3.40 (1H, t, *J* = 9.0 Hz, H-3''), 3.28 (1H, dd, *J* = 11.7, 10.0 Hz, Hb-5''), 3.27 (1H, dd, *J* = 9.0, 8.0 Hz, H-2''), 3.25 (1H, dd, *J* = 9.0, 8.0 Hz, H-2'), ~2.12 (4H, m, H-4 and H-5), 1.68 (3H, br.s, H-10), 1.67 (3H, br.s, H-8), 1.60 (3H, br.s, H-9); ^{13}C-NMR (CD_3OD, 100 MHz): δ 142.0 (C-3), 132.2 (C-7), 124.7 (C-6), 121.1 (C-2), 105.2 (C-1''),102.5 (C-1'), 70.7 (C-4''), 74.5 (C-2''), 77.7 (C-3''), 77.7 (C-3'), 76.5 (C-5'), 74.5 (C-2'), 71.4 (C-4'), 69.2 (C-6'), 66.0 (C-1), 66.6 (C-5''), 39.9 (C-4), 27.0 (C-5), 25.7 (C-8), 17.6 (C-9), 16.2 (C-10). NOESY (CD_3OD, 400 MHz): H-6 ↔ 3H-8. HMBC (CD_3OD, 400 MHz): C3 → 3H-10; C4 → 3H-10; C7 → 3H-8 and 3H-9.

(2*E*)- 3,7-dimethyl-2,6-octadien-1-yl α-L-6-deoxy-mannopyranosyl-(1→6)-β-D-glucopyranoside (10)

^{1}H-NMR (CD_3OD, 400 MHz): δ 5.37 (1H, br.t, H-2), 5.11 (1H, br.t, H-6), 4.75 (1H, d, *J* = 1.7 Hz, H-1''), 4.29 (1H, dd, *J* = 11.8, 6.5 Hz, Ha-1), 4.27 (1H, d, *J* = 7.8 Hz, H-1'), 4.24 (1H, dd, *J* = 11.8, 7.7 Hz, Hb-1), 3.99 (1H, dd, *J* = 11.1, 1.8 Hz, Ha-6'), 3.84 (1H, dd, *J* = 3.5, 1.7 Hz, H-2''), 3.67 (1H, dq, *J* = 9.5, 6.3 Hz, H-5''), 3.66 (1H, dd, *J* = 9.5, 3.5 Hz, H-3''), 3.60 (1H, dd, *J* = 11.1, 6.0 Hz, Hb-6'), 3.37 (1H, t, *J* = 9.5 Hz, H-4''), ~3.36 (1H, m, *sup*, H-5'), 3.33 (1H, m, *sup*, H-3'), 3.29 (1H, m, *sup*, H-4'), 3.17 (1H, dd, *J* = 9.0, 7.8 Hz, H-2'), ~2.13 (2H, m, H-5), ~2.07 (2H, m, H-4), 1.70 (3H, br.s, H-10), 1.68 (3H, br.s, H-8), 1.61 (3H, br.s, H-9), 1.27 (3H, d, *J* = 6.3 Hz, H-6''); ^{13}C-NMR (CD_3OD, 100 MHz): δ 142.2 (C-3), 132.2 (C-7), 124.8 (C-6), 120.9 (C-2), 102.1 (C-1'), 101.8 (C-1''), 77.8 (C-3'), 76.5 (C-5'), 74.6 (C-2'), 73.6 (C-4''), 72.0 (C-3''), 71.9 (C-2''), 71.4 (C-4'), 69.4 (C-5''), 67.8 (C-6'), 65.7 (C-1), 40.3 (C-4), 27.0 (C-5), 25.6 (C-8), 17.7 (C-6''), 17.4 (C-10), 16.3 (C-9). NOESY (CD_3OD, 400 MHz): H-2 ↔ 2H-4; H-6 ↔ 3H-8. HMBC (CD_3OD, 400 MHz): C1 → H-1'; C3 → 3H-10; C4 → 3H-10; C7 → 3H-8 and 3H-9; C6' → H-1''; C-4'' → 3H-6''; C5'' → H-1'' and 3H-6''.
